# A web-based laboratory information system to improve quality of care of tuberculosis patients in Peru: functional requirements, implementation and usage statistics

**DOI:** 10.1186/1472-6947-7-33

**Published:** 2007-10-28

**Authors:** Joaquin A Blaya, Sonya S Shin, Martin JA Yagui, Gloria Yale, Carmen Z Suarez, Luis L Asencios, J Peter Cegielski, Hamish SF Fraser

**Affiliations:** 1 Division of Health Sciences & Technology, Harvard Medical School-MIT, 77 Massachusetts Ave. Cambridge, MA 02139, USA; 2 Partners In Health, 641 Huntington Ave, Boston, MA 02115, USA; 3 Division of Social Medicine & Health Inequalities, Brigham & Women's Hospital, Harvard Medical School, FXB Building, 651 Huntington Ave., 7th Floor, Boston, MA 02115 USA; 4 Instituto Nacional de Salud, Av. Capac Yupanqui 1400, Jesus Maria, Lima, Peru; 5 Direccion de Salud V Lima Ciudad, Jr. Antonio Raymondi 220, La Victoria, Lima, Peru; 6 Direccion de Salud IV Lima Este, Av. Cesar Vallejo s/n, El Agustino, Lima, Perú; 7 Centers for Disease Control and Prevention, 1600 Clifton Rd, Atlanta, Georgia, 30333, USA

## Abstract

**Background:**

Multi-drug resistant tuberculosis patients in resource-poor settings experience large delays in starting appropriate treatment and may not be monitored appropriately due to an overburdened laboratory system, delays in communication of results, and missing or error-prone laboratory data. The objective of this paper is to describe an electronic laboratory information system implemented to alleviate these problems and its expanding use by the Peruvian public sector, as well as examine the broader issues of implementing such systems in resource-poor settings.

**Methods:**

A web-based laboratory information system "e-Chasqui" has been designed and implemented in Peru to improve the timeliness and quality of laboratory data. It was deployed in the national TB laboratory, two regional laboratories and twelve pilot health centres. Using needs assessment and workflow analysis tools, e-Chasqui was designed to provide for improved patient care, increased quality control, and more efficient laboratory monitoring and reporting.

**Results:**

Since its full implementation in March 2006, 29,944 smear microscopy, 31,797 culture and 7,675 drug susceptibility test results have been entered. Over 99% of these results have been viewed online by the health centres. High user satisfaction and heavy use have led to the expansion of e-Chasqui to additional institutions. In total, e-Chasqui will serve a network of institutions providing medical care for over 3.1 million people. The cost to maintain this system is approximately US$0.53 per sample or 1% of the National Peruvian TB program's 2006 budget.

**Conclusion:**

Electronic laboratory information systems have a large potential to improve patient care and public health monitoring in resource-poor settings. Some of the challenges faced in these settings, such as lack of trained personnel, limited transportation, and large coverage areas, are obstacles that a well-designed system can overcome. e-Chasqui has the potential to provide a national TB laboratory network in Peru. Furthermore, the core functionality of e-Chasqui as been implemented in the open source medical record system OpenMRS  for other countries to use.

## Background

Tuberculosis (TB) is a chronic infectious disease that kills over 2 million people per year in the developing world. TB can typically be diagnosed rapidly by sputum microscopy at a local health facility, but diagnosis of multi-drug resistant TB (MDR-TB) – defined as TB strains resistant to at least isoniazid and rifampin – requires a drug susceptibility test (DST) which is usually performed at a regional, national or even supranational level. The emergence of extensively drug-resistant tuberculosis (XDR-TB) heightens the urgency of prompt diagnosis of drug resistance to curb the excessive mortality and ongoing transmission associated with highly resistant strains [1]. Communication of DST results between central and local laboratories and clinical facilities can be problematic and results can take several months [2] to get to their destination or never arrive [3], especially in high-burden countries with limited infrastructure. Prompt treatment with individualized drug regimens based on DST improves patient outcomes [4] and reduces the risk of amplification of drug resistance and ongoing transmission [5,6]. As Raviglione and Smith comment in a recent editorial, "information is essential to build a response [to drug-resistant diseases], and only computerized information systems allow sufficiently rapid exchange of information within and between countries[7]."

### Laboratory Information Systems

Laboratory information systems in developed countries have been shown to decrease turn-around-times (TAT) of laboratory results [8-10], reduce redundancy in resource utilization [9,11,12], and provide faster and more complete notification for public health purposes [13-15]. Shorter TATs have been associated with decreased treatment time, mortality, morbidity, and length of hospital stay [16,17]. We are aware of the use of laboratory systems in the central laboratories in a few developing countries such as Peru and Russia. However, to our knowledge, there are no reports of the use of these systems to link laboratories to clinical settings.

There are potentially greater benefits of using clinical information systems in locations with limited infrastructure where other methods of communications are more costly. However, though they can provide many benefits, these systems are difficult to implement. In developed countries, it is estimated that up to 60% of all information technology implementations in health care fail [18]. Among the many challenges that need to be surpassed are over-burdened laboratory and clinical personnel, frequent staff rotation, limited computer and internet access, and frequent changes in administrations and policies.

### Creating a Peruvian National Laboratory Network

**Figure 1:**
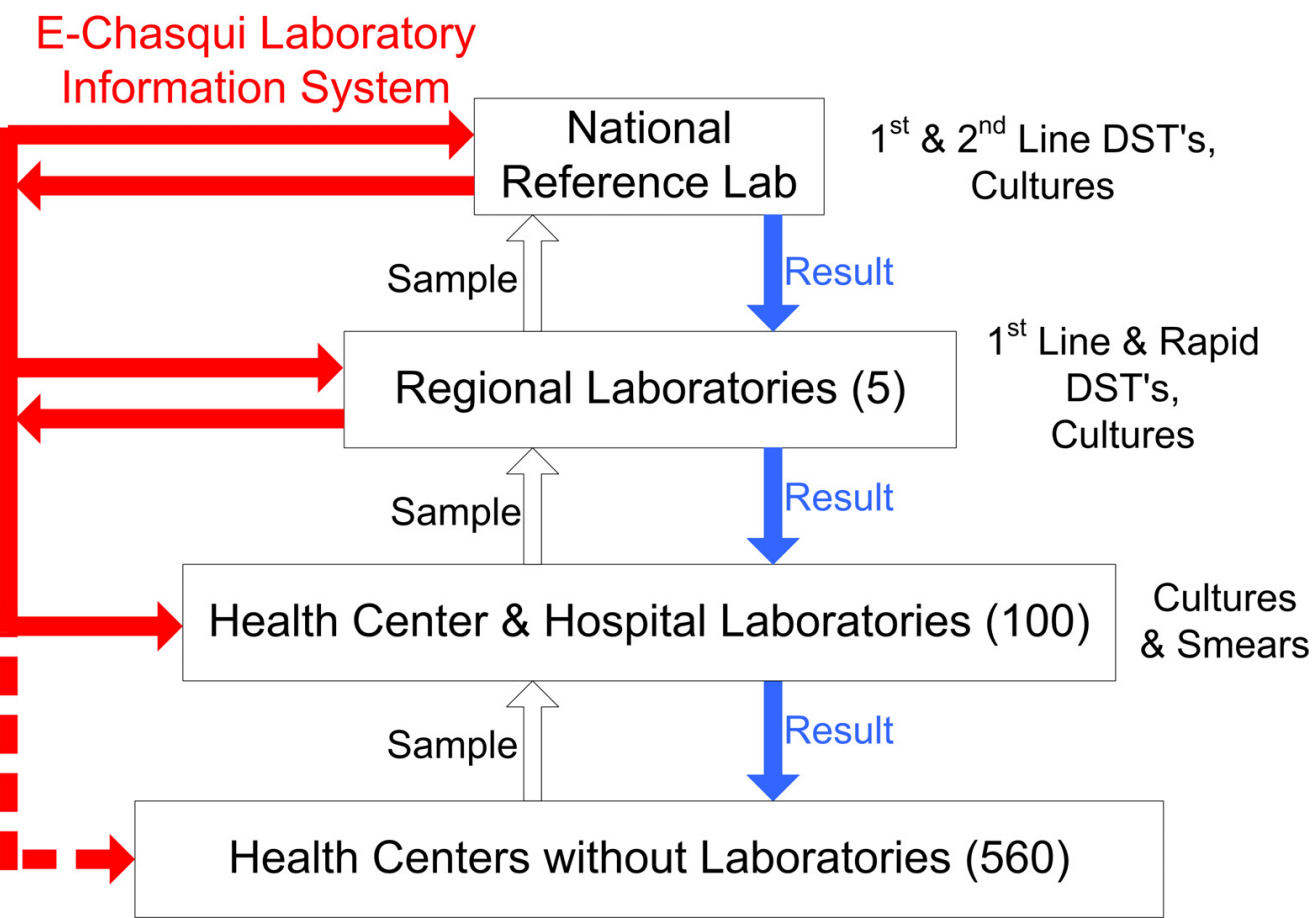
Tuberculosis Laboratory Structure/Workflow in Lima and Locations of e-Chasqui Implementation.

The implementation of decentralized, rapid DST is underway as part of nationwide efforts to scale up services for detection and treatment of MDR-TB and XDR-TB by the Peruvian Ministry of Health [19]. Whereas initially only the Peruvian National Reference Laboratory (NRL) performed DST, the capacity of the regional laboratories has expanded to include rapid and conventional first-line DSTs. The typical flow of a suspected TB patient's sputum sample from the initial treatment site through the laboratory network is depicted in Figure 1. Each test result is communicated serially, and in each step, there are delays and the potential to lose the result.

A study of TATs for cultures and DSTs within the Peruvian public health system suggests that patients could still experience risky delays despite availability of decentralized, rapid DST unless programmatic aspects are also addressed [2]. In addition to reducing communication delays, minimizing lost and erroneous results is essential for reducing morbidity in these high-risk patients. To improve these aspects, we developed and implemented the laboratory information system described herein.

This paper describes the design and implementation of a web-based TB laboratory information system to communicate data between a national laboratory, two regional laboratories, and 12 health centres (HC) in Lima, Peru. This system was designed to support a national TB laboratory network connecting all participating institutions. We then describe the expansion of the system at the request of the public administration. Finally, we examine broader issues of implementing these types of systems in resource-poor settings including costs and sustainability.

## Methods

### Needs Assessment

The first step in creating the laboratory information system was to conduct a needs assessment of the major stakeholders: the personnel in the HCs, regional, and national laboratories. After working with the director, laboratory technician and data entry staff in the participating laboratories and the TB clinician, nurse and local laboratory technician in several key HCs, a list of information requirements was created, shown in Table 1. While most requirements were identified during this initial period, others emerged during the implementation process.

**Table 1:** Needs Assessment of Health Centres and Laboratories

**Health Centres**
All information displayed to mirror paper forms
Find patient by name despite constant misspellings
Fast access despite low bandwidth
Easily access patient's individual result and history of all results
For a sample view all tests performed and date when sample was taken
View all recent results by HC
Track all tests pending by HC
Access information on samples collected in other institutions (e.g. while hospitalized, prior to transfer to their HC)
Email notification of new test results
Print out a test result in the official MINSA format
Display trend in DST requests by HC
Show MDR-TB patients not appropriate treatment
Current patients failing treatment
Access latest information on evidence-based TB treatment
**Laboratories**
Integrate into laboratory workflow with minimal disturbance or increased work
Search for sample by ID number
Individual results printed in current paper form
Aggregate reporting for all tests entered
Ability to view all culture and DST results reported within an arbitrary time period
Improve quality control of test results
Ability to modify or "grow" system with continual requirements
Compatibility with existing computerized information systems

### Integration into Laboratory Workflow

The laboratory information system needed to be integrated within the workflow of the busy regional and central laboratories. We performed a thorough workflow analysis of each laboratory's systems of information, each staff's responsibilities, quality control, and tests performed, and designed the system to follow the current workflow of intake, processing, and reporting. However, the integration of the information system still required workflow adjustments to incorporate data entry, digital verification, and printing of results from the system. This was done through iterative discussions with the laboratory directors followed by an hour-long training session for all laboratory personnel. These changes in workflow, however, did not result in increased time demands; instead the revised system resulted in greater efficiency for most laboratory personnel, since the database (with reliable back-up) obviated the need to photocopy and maintain physical copies of all results at the laboratory.

Finally, the laboratory information system had to integrate with current laboratory reporting systems being used. During the implementation of e-Chasqui the NRL moved from using the PHLIS laboratory reporting system [20], to an in-house developed laboratory management system. To communicate data between these systems, a tool was created to manually export all results; we are currently defining other inter-system communication methods.

### System Design

The electronic laboratory information system, called e-Chasqui, supports the decentralized entry and viewing of bacteriological tests (smear microscopy, cultures, species identification, and DSTs). The Chasquis were agile and highly-trained runners that delivered messages, royal delicacies and other objects throughout the Inca Empire and are a source of pride in Peru. In addition, it includes applications to assess quality control, generate aggregate reports, notify health centres of new results or contaminated samples, and track both enrolled patients and the status of pending laboratory tests. e-Chasqui extends the web-based TB electronic medical record system, PIH-EMR, that has been in use in Peru since 2001 [21,22]. To protect patient's confidentiality, e-Chasqui incorporated extensive encryption and web security features for medical records of the PIH-EMR [23]. Furthermore, all users sign a confidentiality agreement before being given access.

We worked with the national and regional district and laboratory directors to define the access profiles for the different types of users. Clinical personnel have individual access to all patients under their responsibility e.g. single HC, multiple HCs, or a full district. Examples of clinical personnel include HC staff, the regional TB program director, and the regional treatment approval committees, composed of pulmonologists and clinicians. Laboratory personnel have both an individual and aggregate view of laboratory test results. Defining the types of access, getting all stakeholders to agree, and building the flexibility into the system was one of the most difficult tasks in building e-Chasqui.

The ultimate goal of the system is for all laboratories, including those at HCs, to enter tests they've performed and use the system to order further tests. However, in the initial phase all data was entered at the NRL and regional laboratories with "read-only" access provided to HCs. Therefore when the first e-Chasqui laboratory receives a sample, they enter all previous test results performed on that sample.

#### Patient Care

**Figure 2:**
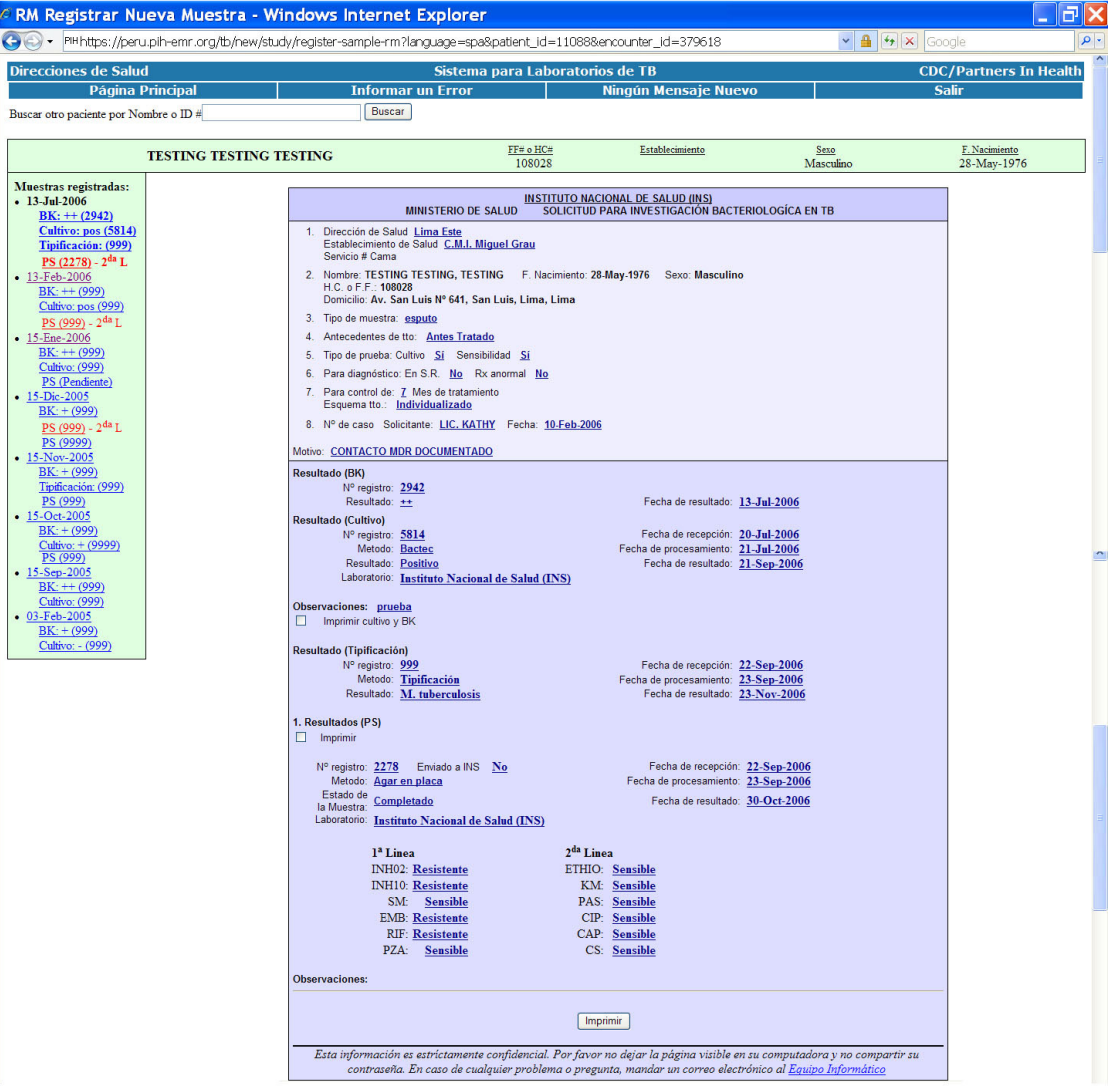
**e-Chasqui main patient page**. This page shows the patient's full bacteriological history on the left sidebar and with bolded sample date for the sample whose results were being displayed on main part of page.

The core of the e-Chasqui interface is a single patient page containing the history of all tests performed for the patient on a left sidebar, and the details for any single sample on the main part of the page (Figure 2). For a single sample, tests can be performed by up to four different laboratories. All test results are displayed in this single page to give the full history of the sample. This novel tracking ability is a useful addition; prior to e-Chasqui's implementation, laboratory and clinical personnel systems lacked the test request date or the smear or culture data when they received a DST result. The system uses a flexible search algorithm by either the patient's names (including partial names) or by any of the sample's test identification numbers. This patient page, like all others, contains only text and uses optimized SQL queries to load quickly even in areas with low bandwidth.

From this page, the user can select which tests to print in the official report format. Though each HC can print the report immediately after laboratory verification, each laboratory also prints a copy and sends this stamped "official" report to the HC for their paper records. Due to the high load of TB patients, the HC personnel requested the ability to view their latest results on a single page and track the status of all their samples being processed. Tools were designed to meet these requirements. Finally, all HC users receive nightly email notifications for new test results on patients attending their HC.

#### Laboratory Quality Control

The laboratory personnel described long-standing problems with ensuring the timeliness of reporting results. Since a culture or DST result takes 20 to 60 days to be read, some tests "fell through the cracks" and were not read, or were read late. Furthermore, they also requested ways to ensure all results had been entered, to minimize duplicate tests, and to monitor the contamination rate. Therefore, the system was expanded to incorporate quality control tools to remind personnel to read samples on a regular basis, flag duplicate or missing results, and report contamination rates. These tools are usually automatically updating tables or lists of tests that show the appropriate information.

#### Laboratory Monitoring/Reporting

An initial reporting tool was created for the regional laboratories to view all results. Further monitoring and reporting tools were created as the needs arose throughout the implementation process. They can be seen in Table 2.

**Table 2:** Reports generated by e-Chasqui

**Report**	**Informed**	**Purpose**	**Type of Access**
Frequency of e-Chasqui access by HC personnel	Regional laboratory and TB director	Encourage frequent utilization of IS to access real-time laboratory data	Monthly report prepared by data administrator
Number of laboratory results entered at regional laboratory	Regional laboratory and TB director	Identify delays in data entry	Monthly report prepared by data administrator
Number of laboratory results verified and released to providers	Regional laboratory and TB director	Identify delays in verification	Monthly report prepared by data administrator
DST results for any specified period grouped by every variable in request form	Regional and INS laboratory director	Report and identify trends in laboratory performance	Constant access**
Culture results for any specified period grouped by every variable in request form	Regional and INS laboratory director	Report and identify trends in laboratory performance	Constant access**
Individuals with a positive culture for any specified date	Regional and INS laboratory director	Report to regional TB program	Constant access**

**Constant access means that the laboratory users could view this information in the system at any time. Some reports let the user specify the start and end dates.

### Implementation

Though described separately from the needs assessment and system design, the deployment of e-Chasqui in the laboratories and HCs was complementary and overlapped as the use and functionality of e-Chasqui grew.

#### Information Technology Assessment

The initial step of implementation consisted of an assessment of the information technology status at each HC and laboratory, performed by the regional health districts, and included data such as the number and condition of computers in each HC, physical security, and internet access. The assessment identified key deficits, and we were able to coordinate with each health district to perform corrections such as donating or fixing computers and providing or improving internet access.

#### Laboratories

The commitment of the health districts was demonstrated by providing a part-time data entry person specifically for e-Chasqui. We trained all laboratory staff in the workflow changes and in the use of e-Chasqui during a single 1-hour group training session. We also had individualized sessions for each user since each had different responsibilities, on average lasting approximately 1.5 hours. After several months of use, two of the three laboratories requested that the technicians also have e-Chasqui access.

For data entry several simple design tools were implemented and found to be valuable. First, for ease of data entry each data field can be accessed not only by clicking on the field with the mouse, but also by sequential tabbing through the page. Second, the main patient page was identical to the test request form from which the data entry occurred. To avoid duplicate patients when a new patient is being created, e-Chasqui searches for patients with similar names, and if any are found a warning is displayed where the user can click on one of the existing patient names or click the "Create New Patient" button. Also, a tool to merge patient records was created to handle duplicates. Duplicate sample records are handled using data quality tools, explained previously in the Laboratory Quality Control section

The system had to be continually expanded and adapted to the needs encountered during the pilot phase. During the first eight months after implementation, functionality to generate lists of reported DSTs and the quality control tools were created. In the following 3 months, we added pages for the HC users to view the tests currently being processed and a consolidated view of the last 3 weeks of results. In Sept. 2006, 11 months after initial implementation, the NRL began to use this system required changes to accommodate its specific workflow. At the same time we modified the system, at HC users' request, to send only one email at night if results had been verified that day, as opposed to an email for every result verified.

#### Health Centres

Once a HC had a computer with internet access that could be used by the TB personnel, all users were trained in a single 1-hour session in computer use, confidentiality procedures, and use of e-Chasqui. The e-Chasqui data administrator then performed follow-ups every third week. In most HCs, we identified at least one "champion" who uses the system frequently. However, rarely did we find this champion promoting the system to others.

Throughout the implementation, we had to troubleshoot problems. Most of the problems were administrative or hardware related such as having to create a new windows XP user, ensuring that HC users were viewing their results in e-Chasqui in a timely fashion, replacing a stolen computer, and providing six web access points to TB programs within HCs that lacked computer access (Baobab Health Partnership) [24].

## Results

The needs assessment and workflow analysis began in June 2005, with the first user testing in July 2005, January 2006, and May 2006, for each of the two regional and the national laboratories, respectively. Full implementation occurred in March 2006, August 2006, and September 2006, respectively.

### System Usage

**Figure 3:**
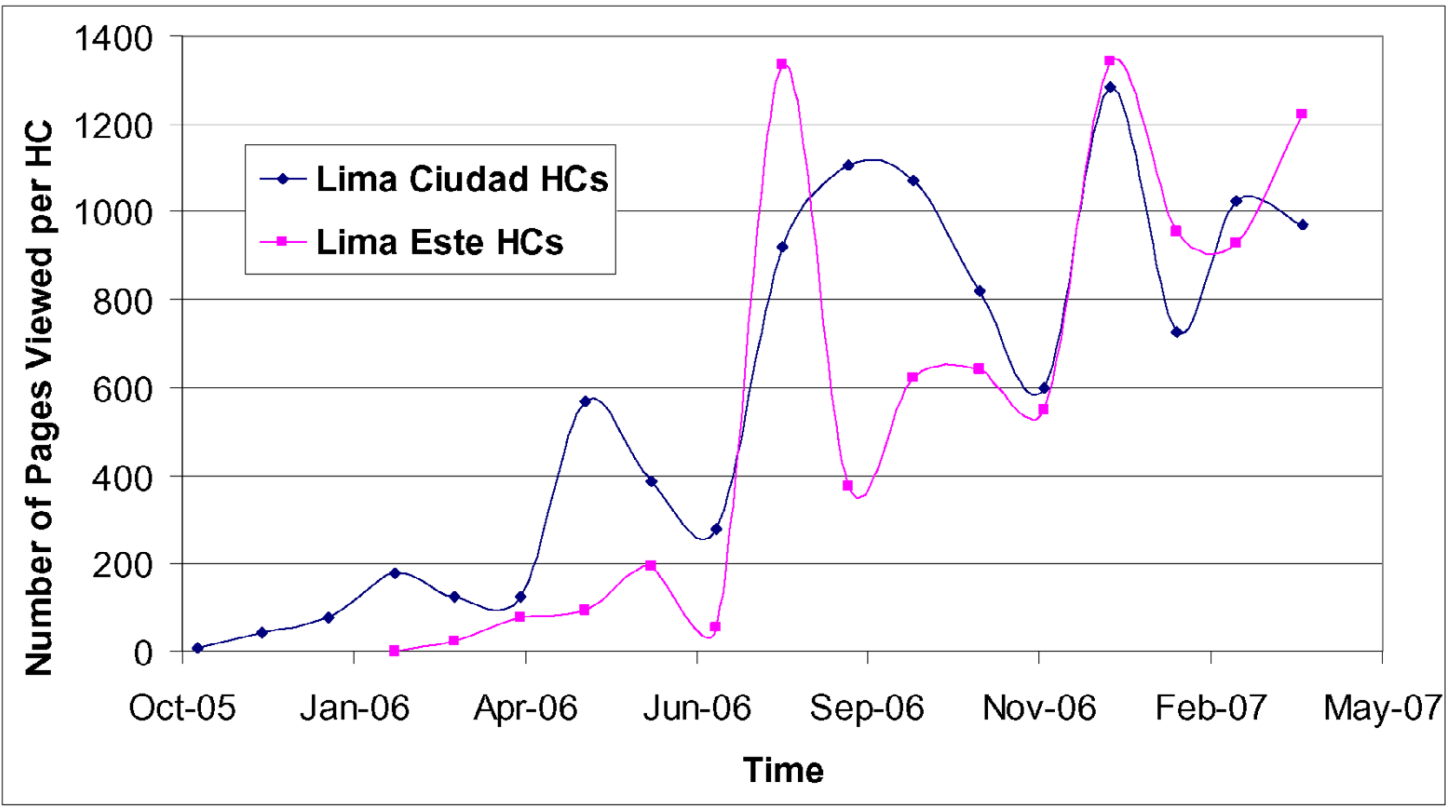
**Average monthly number of pages viewed by health centres (HC)**. The average number of pages viewed by the HCs in each of the two health districts (Lima Ciudad, Lima Este) where e-Chasqui is implemented. Full implementation occurred in March 2006 (Lima Ciudad) and August 2006 (Lima Este).

Our system has been successfully integrated into program operations. Since its initial implementation, 29,994 smear microscopy, 31,797 culture and 7,675 DST results have been entered. In 2006, 99.5% of all DST results and 98.8% of all culture results for the 12 pilot HCs were viewed online. The average number of pages viewed by the HCs in each of the two health districts (Lima Ciudad, Lima Este) can be seen in Figure 3. The large increase in pages viewed in August 2006 occurred because e-Chasqui was fully implemented in both the Lima Este regional laboratory and the NRL.

This is an online transaction processing system and since it is used in sites with low to medium internet bandwidth, this is a major factor in its performance. Due to e-Chasqui's simple, text-based design all sites can use it during routine clinical and laboratory work. In 2006, the system performed on average 1865 transactions per day including page views, data entry, and analysis. In 2007, it has increased to 4501 daily transaction and the system's performance has not been appreciably affected. Feedback from users has been positive. This feedback has been in the form of conversations by the research staff with the clinical and laboratory personnel, increased usage of the system by intervention sites, and requests for expansion of the use of the system by the district and laboratory administrators. Importantly, we have been careful to respond to critical comments and suggestions to enhance the system and maintain user "buy-in." A strong indicator of the system's utility is that district administrators have requested expansion of the system to additional institutions. In response, we are expanding access to three laboratories, 2 hospitals and 11 HCs that administrate 47 other health centres. In total, e-Chasqui will serve a network of institutions providing medical care for over 3.1 million people.

### System Costs

In quantifying the costs of designing and implementing this web-based system in Peru, we have found the annual recurring cost to be US$34,738 total or US$0.53 per sample entered. More details can be found in Table 3. This figure includes the cost of full internet access to all e-Chasqui institutions and a US based system manager. Since HCs use the internet for other purposes, including the national health register, we feel the system should incur 50% of the internet cost. Also, if the system manager was Peruvian with a local salary, the annual recurring cost reflects the approximate cost of implementing e-Chasqui in all major health centres in the two health districts. For comparison, the e-Chasqui health districts had 1103 MDR-TB patients on standardized or individualized treatment in 2006. The annual cost of these treatments are approximately US$2,900 and US$3,000, respectively [25]. Another comparison is that these health districts accounted for 53% of TB and MDR-TB patients in a national program whose 2006 budget was close to US$10 million [26]. In either case, this system to communicate all vital laboratory data for TB and MDR-TB treatment accounted for approximately 1% of the budget for those districts.

**Table 3:** Fixed and Monthly Costs of implementing e-Chasqui

	**Calculation**	**Fixed Cost**	**Monthly Cost**
**Infrastructure Building**			
Computers, web access points and installation	8 × $458 (average cost)	$3,666.00	
Printers	4 × $150	$600.00	
Server		$2,500.00	
Internet for health centres and labs	12 HCs & 2 labs × $41 monthly		$574.00
Internet for headquarters with server	1 HQ × $400 monthly		$400.00

Total		$6,766.00	$974.00

**System Design & Development**			
Peruvian Clinician	80 hours × $21/hour	$1,680.00	
System Manager	500 hours × $22/hour	$11,000.00	
Faculty Consulting	40 hours × $59/hour	$2,360.00	
Programmer	100 hours × $40/hour	$4,000.00	

Total		$19,040.00	

**System Implementation**			
System Manager	620 hours × $22/hour	$13,640.00	
Faculty Consulting	80 hours × $59/hour	$4,720.00	
Programmer	450 hours × $40/hour	$18,000.00	

Total		$36,360.00	

**Data Entry & Management**			
System Manager	1/4 time		$937.50
Peruvian Data Administrator	2/3 time		$253.33
Peruvian Data Entry (one per lab)	3 × 2/3 time		$580.00
Transportation for Data Administrator	1.5 monthly visits to every site		$150.00

Total			$1,920.83

**System Advocacy**			
Peruvian Clinician	100 hours × $21/hour	$2,100.00	
Faculty Consulting	50 hours × $59/hour	$2,950.00	
System Manager	200 hours × $22/hour	$4,400.00	

Total		$9,450.00	

**Grand Total**		**$71,616.00**	**$2,894.83**

We have divided the costs into five categories: infrastructure building, system design and development, system implementation, data entry and management, and system advocacy. For infrastructure, the objective is to have every health institution with a computer, printer and intermittent, if not constant, internet connection. System advocacy has consisted of meetings and discussions, usually with national or regional administrators, to discuss the system's potential benefits, provide updates on its status, and train users on the system's abilities since this was the first time a web-based clinical system had been implemented. The costs incurred by a new program implementing e-Chasqui should be reduced as they will not include system development. All costs are in 2007 U.S. Dollars Unless explicitly stated all staff are US based

## Discussion

### Challenges and Obstacles

#### Creating a system with enough flexibility to meet all stakeholders' needs that arise during implementation

Though e-Chasqui has focused functionality, the need to create many types of users and to define methods of communication between institutions took much work and time. There were two main reasons for this. First, the inexperience in implementing clinical information systems among stakeholders meant much learning about this topic had to take place. As a result, the technical requirements of e-Chasqui were constantly revised. For example, some stakeholders were unfamiliar with the concept that different users see information in specific manner such as individual or aggregate views. Therefore some exhibited initial scepticism about the system's ability to maintain information confidential. Second, defining appropriate user accesses to balance patient confidentiality with users' request for information. Again due to e-Chasqui's novelty, both the developers and the institutions have had to learn what the appropriate user permissions were. Here the web-based architecture allows e-Chasqui to track all users' actions. This capability was highly valued by all stakeholders since many of them asked about data confidentiality and security.

#### Maintaining both high data quality and timeliness with limited staff

The balance between opportune entry of results and electronic verification with high data quality continues to be a problem. The mean number of days between a DST result being read, its entry, and verification is 5.8. Though we believe that the additional step of result verification ensures higher data quality, we are still working to minimize these delays. On the other hand, the average number of days from laboratory verification to the HC personnel viewing their result in e-Chasqui is 2.2 which shows their interest in updated results.

#### Strengthening public infrastructure

To ensure e-Chasqui had lasting impact on patient care, it was necessary to integrate this system within the public health structure. Though this can mean additional work in terms of agreements with the different national and regional institutions, as well as providing additional services, the long lasting benefits, such as sustainability and implementation at a national level, usually outweigh this additional work.

### Lessons learned

TB programs trying to improve communications, monitoring, and patient care by implementing electronic information systems face a task that can sometimes seem overwhelming. We have learned several lessons from our experience developing a nation-wide electronic laboratory information system in Peru.

#### All important stakeholders must contribute to the design and implementation

This is the only way to ensure the system addresses the actual user needs and to have user appropriation. To identify key system attributes during the design, medical and laboratory personnel must be involved from the beginning. Furthermore, developers must create a system easily integrated into the existing workflow with minimal disruption and sufficient advantages to gain "buy-in" such as easy usage for people with little computer experience. Lastly, branding the system appropriately, perhaps with a familiar name, makes it more recognizable. During the system's implementation, users must be constantly asked if they have questions or problems and their suggestions for fixing them. Problems that are outside the system's scope, such as not having access to a computer with internet, personal conflicts with other personnel who would like internet access, or equipment failures, should be addressed with administrative personnel.

#### Political support is integral to the system's dissemination

Unless there is will from the administration to implement an electronic information system, promote its use, and allocate resources to maintain it, there is little chance of success. This system was implemented as part of a scale up strategy between the National Tuberculosis Program and NRL to expand the laboratory network. Political support in this case was demonstrated by the support of the regional health administration and by laboratories providing data entry staff.

#### Provide adequate training in the system's use and benefits

Training should be focused on the benefits that it provides to the users. In Peru, most previous health information systems have required HC personnel to enter data for reporting purposes without receiving any feedback. While implementing e-Chasqui, we saw reticent users become enthusiastic when they realized the system would provide *them *with useful information. Training must also be provided continually, and the system's use monitored to ensure it continues to meet user's needs.

#### Ensure the system's sustainability

Sustainability in our experience is maintained by generating user confidence in the system's quality and usability, creating a flexible system able to adapt to changes within the public system, and providing evidence of system benefits. To have user confidence, the system must actually save time and be perceived as a *consistently *useful tool after the initial novelty has worn off. Three main factors to promote sustainability include (1) providing and maintaining a functional internet access point at their HC, (2) ensuring the quality and promptness of data, and (3) providing support to all users. Support to all users usually took the form of technical assistance at the laboratories and up-to-date results to HCs.

#### Implement the system as part of a larger structural improvement

We believe that the implementation of an information system is enhanced if it is an integral part of larger improvements in the clinical or laboratory infrastructure. That way the system can not only help improve communication but also be part of a more general improvement in workflow. In the case of e-Chasqui, it was incorporated into national project to decentralize DSTs.

## Conclusion

Electronic laboratory information systems have much potential to improve patient care and public health monitoring in resource-poor settings. Some of the challenges described, such as lack of trained personnel, limited transportation, and large coverage areas, are obstacles that a well-designed information system can overcome. However, creating well-designed information systems is a difficult task necessitating appropriate resources, expertise and time to be successful.

The purpose of this paper is to pass on our experience of critical design issues and required capabilities to make similar systems work on-site. Though other projects will need to design and rollout laboratory information systems, we hope to make the process less onerous next time around.

e-Chasqui has the potential for creating a national TB laboratory network in Peru to facilitate the communication and analysis of all bacteriological results country-wide. We have already begun to see additional benefits to this system such as having the test always available during clinical decision making, reducing duplicate tests performed, and reducing the time and money spent by staff checking the status of their samples. Studies have been initiated to quantify these benefits. We are also conducting a prospective and retrospective evaluation study to measure e-Chasqui's effect on reducing mean delays, "lost" results with excessive delays, and errors of laboratory reporting. Furthermore, this same system or one similar could more easily be implemented in other countries facing similar problems of test tracking. In our efforts to make these systems available, we are implementing the core functionality of e-Chasqui as a module in the OpenMRS system [27,28]. OpenMRS is a general purpose medical record system architecture we have developed with colleagues in the US and Africa to support TB and HIV treatment programs. OpenMRS is being rolled out in eight countries[29] with support from the US Centers for Disease Control and Prevention and the World Health Organization. At least two of these countries will use the e-Chasqui component.

## Competing interests

The author(s) declare that they have no competing interests.

## Authors' contributions

JB carried out the design and implementation of the system and drafted the manuscript. SS conceived of the system and helped to draft the manuscript. MY participated in the implementation of the system, its expansion and helped to draft the manuscript. CS, GY and LA carried out the use of the system in the laboratories and health centres in Lima. PC participated in the initial design of the system and the evaluation methodology. HF participated in the design and implementation of the system and helped to draft the manuscript. All authors read and approved the final manuscript.

## Pre-publication history

The pre-publication history for this paper can be accessed here:


